# Shear Bond Strength of Orthodontic Brackets on Demineralized Enamel Before and After Application of a Resin Infiltrant Remineralizing Agent: An In Vitro Study

**DOI:** 10.3390/dj14050299

**Published:** 2026-05-14

**Authors:** Ahmed Almahrul, Ikuo Yonemitsu, Tomoko Tabata, Masaomi Ikeda, Yuka Tanaka-Takemura, Yasushi Shimada, Takashi Ono

**Affiliations:** 1 Department of Orthodontic Science, Graduate School of Medical and Dental Sciences, Institute of Science Tokyo, 1-5-45 Yushima, Bunkyo-ku, Tokyo 113-8510, Japant.ono.orts@tmd.ac.jp (T.O.); 2 Department of Cariology and Operative Dentistry, Graduate School of Medical and Dental Sciences, Institute of Science Tokyo, 1-5-4 Yushima, Bunkyo-ku, Tokyo 113-8510, Japanshimada.ope@tmd.ac.jp (Y.S.); 3 Department of Oral Biomedical Engineering, Graduate School of Medical and Dental Sciences, Institute of Science Tokyo, 1-5-45 Yushima, Bunkyo-ku, Tokyo 113-8510, Japan; ikedsdt@tmd.ac.jp

**Keywords:** shear bond strength, swept-source optical coherence tomography (SS-OCT), demineralization, resin infiltrant, in vitro study, orthodontic brackets

## Abstract

**Background/Objectives**: We evaluated whether resin infiltration treatment of demineralized enamel improves shear bond strength (SBS). **Methods**: Thirty permanent bovine incisor teeth were assigned randomly into three groups (*n* = 10 per group): control group, demineralized enamel pretreated with ICON^®^ resin infiltrant (Exp1 group), and demineralized enamel without pretreatment (Exp2). Demineralization was induced using a pH 4.5 solution for 21 days and was monitored using swept-source optical coherence tomography on days 0, 7, 14, and 21. The lesion depth (LD) was quantified and evaluated using ImageJ software. In the Exp1 group, ICON^®^ was applied prior to bracket bonding; no pretreatment was applied in the Exp2 group. In all groups, brackets were bonded using Super-Bond/Clear fluoride-free self-cure adhesive resin (4-META/MMA-TBB, Sun Medical) following Phosphoric acid (65%; Red Activator, Sun Medical). After debonding, enamel surfaces were evaluated to determine the adhesive remnant index (ARI). **Results**: No significant difference (*p* = 0.631) was noted in LD between Exp1 and Exp2 groups. The SBS values significantly differed (*p* < 0.05) between the control (4.1 ± 1.0 MPa) and Exp1 (5.5 ± 1.4 MPa) groups and between the Exp1 and Exp2 (3.8 ± 1.3 MPa) groups. However, SBS did not differ significantly between the control and Exp2 groups. Furthermore, ARI scores showed no significant difference between the control and Exp1 groups, whereas the Exp2 group recorded significantly elevated ARI scores relative to the control group (*p* = 0.0127). **Conclusions**: These findings suggest that resin infiltration with ICON^®^ may improve bracket adhesion on demineralized enamel.

## 1. Introduction

Several studies have demonstrated that oral hygiene tends to rapidly deteriorate once a patient begins fixed orthodontic treatment [1,2,3,4]. A major complication associated with orthodontic appliances is demineralization of the enamel [5]. If oral hygiene is inadequate during fixed orthodontic treatment, biofilm accumulation areas are created, which encourage the initiation of white spot lesions (WSLs), especially in individuals with elevated carbohydrate intake [6,7]. The retained biofilm exhibits an increased acidogenic potential on the enamel surface, which causes prolonged periods of low pH [8]. When fluoride supplementation is absent, enamel mineral loss around fixed orthodontic appliances can advance quickly, with subsurface lesions becoming detectable within weeks of appliance placement [9]. Clinical studies have confirmed that early enamel demineralization progresses rapidly if preventive interventions are not implemented [10]. At least one WSL was visible in 38% of the patients after 6 months and in 46% after 12 months of fixed orthodontic treatment; both rates were markedly elevated compared with those in the control group [11]. After 6 months of observation, several new enamel demineralization lesions appeared in orthodontic patients, including on vestibular and proximal tooth surfaces near the brackets [12].

New WSL formation is most rapid within the first 6 months of orthodontic treatment, after which the rate of lesion development tends to stabilize around 12 months [13]. Adequate plaque removal is considerably challenging once fixed orthodontic appliances are in place, as brackets and archwires create additional retention sites that predispose enamel to demineralization [14]. Another study demonstrated that orthodontic patients experienced higher rates of WSLs than non-orthodontic individuals, with prevalence rates ranging from 50% to 90% [15].

Among available strategies for WSL management, the application of low-viscosity photopolymerizable infiltration resins has emerged as an effective option. Infiltrants have high surface tension and very low viscosity; thus, they offer a minimally invasive option for managing early carious lesions [16]. Certain studies have demonstrated that these resins infiltrate enamel lesions more deeply than conventional adhesives [17] and may outperform fluoride-based materials in inhibiting lesion progression [18].

Adequate SBS is a fundamental requirement for successful orthodontic bracket retention. Clinical bracket retention requires a minimum SBS within the range of 5.9 to 7.8 MPa, based on in vitro literature, to resist the forces generated during orthodontic treatment [19]. However, bond strength values that are excessively high can compromise enamel integrity at the time of bracket removal, and it is generally recommended that SBS remain below 10 MPa to reduce the risk of enamel damage [20,21]. Bovine teeth are widely used as human tooth substitutes in laboratory SBS investigations, primarily because of their availability and the ethical limitations associated with obtaining extracted human teeth [22,23,24].

The presence of enamel demineralization adversely affects orthodontic bracket bonding. Substantial bodies of in vitro literature demonstrate that bracket adhesion is considerably weaker on demineralized enamel than on structurally intact tooth surfaces [25,26]. This weakening is explained by the altered microstructure of demineralized enamel, which impairs the formation of normal acid-etching patterns and disrupts the mechanical coupling at the enamel–adhesive junction [27,28]. Furthermore, beyond reducing adhesive retention, the structurally weakened enamel associated with mineral loss also raises the risk of enamel damage occurring when brackets are subsequently removed [29].

In addition to arresting lesion development, resin infiltration improves esthetics [10]. The increased porosity of demineralized enamel facilitates acid diffusion; however, infiltrating resins can occlude these pathways and halt further demineralization [17]. ICON^®^ is a commercially available resin infiltration system. This treatment was found to improve microhardness compared with that of untreated demineralized enamel [30]. Clinical evaluations have demonstrated significant masking of WSLs following resin infiltration, with reductions in the lesion area and stable esthetic outcomes at follow-up [31]. Different studies have demonstrated that this treatment is minimally invasive, preserves enamel integrity, and can be combined with orthodontic adhesives without compromising bond performance [30,32,33].

Because WSLs are frequently encountered during orthodontic treatment, the use of a resin infiltrant, such as ICON^®^, may be effective in halting lesion progression while maintaining sufficient bond strength for orthodontic brackets [34]. To our knowledge, the present study is the first to use swept-source optical coherence tomography (SS-OCT) for longitudinal monitoring of enamel demineralization depth while concurrently evaluating the effect of ICON^®^ resin infiltration on orthodontic bracket SBS. Mineral loss detected by SS-OCT is reflected by an increased signal intensity and corresponding alterations in the brightness profiles according to the degree of demineralization. These findings indicate that SS-OCT has demonstrated considerable potential as a non-invasive diagnostic tool for tracking the development of early enamel carious lesions [35].

The present study was therefore designed to determine whether resin infiltration of demineralized enamel enhances bracket SBS and to characterize the associated failure mode distribution and lesion depth changes through serial SS-OCT imaging.

## 2. Materials and Methods

### 2.1. Specimen Preparation

**Figure 1:**
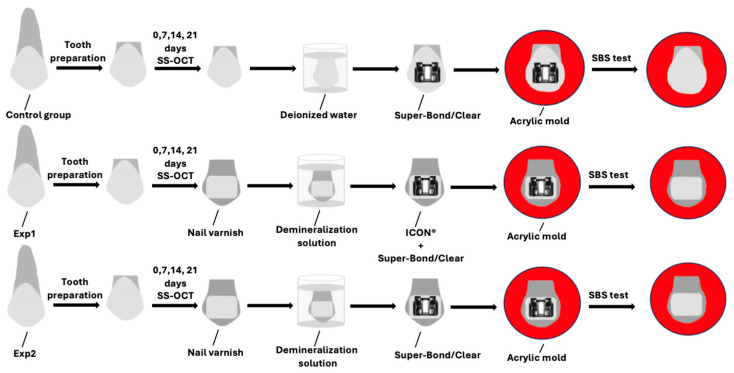
Diagram illustrating the overall experimental design and procedural steps.

An overview of the experimental design is provided in Figure 1.

A total of thirty permanent bovine incisors were procured, assessed for any damage sustained during extraction, cleaned carefully, rinsed under running water, and kept at 0 °C until use. The crowns were sectioned using a precision diamond saw operating at low speed (Isomet; Buehler, Lake Bluff, IL, USA). The labial enamel surface at the mid-coronal level was then flattened by sequential abrasion with silicon carbide papers of ascending grit sizes (600–2000; Fuji Star, Sankyo Rikagaku, Saitama, Japan). The enamel surface was ground to obtain a standardized flat surface, as described in previous in vitro studies [36]. The prepared specimens were randomly assigned to three experimental groups (*n* = 10 each). Following preparation, specimens were distributed randomly into three study groups (*n* = 10 per group). This group size was determined by reference to a comparable previously published in vitro SBS study that used bovine teeth with equivalent sample numbers [37]. Baseline SS-OCT measurements were obtained from the enamel before demineralization in the three experimental groups. For the demineralized enamel pretreated with ICON^®^ resin infiltrant (Exp1 group) and the demineralized enamel without pretreatment (Exp2 group), nail varnish (Revlon Nail Enamel, Revlon, New York, NY, USA) was painted onto the enamel surface, leaving an uncovered area of 5 × 4 mm^2^ on each crown to act as the zone targeted for demineralization. The surrounding enamel was fully coated to serve as a reference area for comparison.

### 2.2. Demineralization Protocol

The demineralization solution consisted of 2.2 mmol/L CaCl_2_, 2.2 mmol/L NaH_2_PO_4_, and 50 mmol/L acetic acid, adjusted to pH 4.5 with NaOH [38,39,40,41]. For the control group, the specimens were immersed in deionized water, which was replaced every 24 h, and incubated at 37 °C for 21 days. In contrast, specimens in the Exp1 and Exp2 groups were immersed in the demineralization solution under continuous vibration for 21 days, and the demineralization medium was freshly renewed each day.

### 2.3. SS-OCT System

The SS-OCT system (OCT-2000; Santec, Komaki, Japan) incorporated a high-speed swept-source external-cavity laser operating across a wavelength range of 1260–1360 nm, centered at 1310 nm, with a sweep repetition rate of 20 kHz. The backscattered light from the specimen was coupled back into the SS-OCT system, where the resulting interference signal was recorded as a function of time and transformed via Fourier analysis to yield spatially resolved depth information. The system achieved lateral and axial resolutions of 17 µm and 11 µm in air, respectively, corresponding to an axial resolution of approximately 7 µm within tissue when a refractive index of 1.5 is assumed. Complete B-scan image acquisition required 0.3 s, inclusive of processing. Each image covered a field of 5 mm (width) × 6.6 mm (height) and comprised 2000 × 1019 pixels. The measured system sensitivity and shot-noise-limited sensitivity were 106 dB and 119 dB, respectively. Each specimen was inclined at an angle of 3–5° relative to the incident beam to eliminate specular surface reflections from the captured images. SS-OCT measurements were performed starting on day 0 and continued on days 7, 14, and 21 in all groups. A purpose-written analysis routine within ImageJ (version 1.48, National Institutes of Health, Bethesda, MD, USA) was employed to import and process the raw SS-OCT datasets. Reconstructed images were rendered at a magnification ratio of 3:1, corresponding to physical dimensions of 5000 × 7481 µm. A threshold plug-in was applied, and a region of interest (ROI) measuring 100 × 100 µm (333.33 × 748.1 µm in the scaled image) was randomly selected and marked before nail varnish was applied. The ROI was selected 50 µm away from the nail varnish area to minimize potential backscattering artifacts in the SS-OCT measurements.

### 2.4. Application of ICON^®^

In accordance with the manufacturer’s protocol, the enamel surface was conditioned with 15% hydrochloric acid gel applied for 2 min, after which the surface was thoroughly washed with water for 30 s. The desiccation step was then performed using Icon-Dry (99% ethanol) for 30 s, followed by gentle air drying. ICON^®^ resin infiltrant (DMG; Hamburg, Germany) was subsequently delivered onto the conditioned surface and allowed to penetrate for 3 min before being light-cured for 40 s with an LED curing device (Pencure 2000; Morita, Osaka, Japan) set to an output of 800 mW/cm.

### 2.5. Bonding Procedure

A lower incisor plastic bracket (Esther MB; Tomy, Fuchu-shi, Tokyo, Japan) was centered on the prepared enamel surface, and the adhesive was dispensed onto the bracket base. Enamel etching was performed by applying 65% phosphoric acid (Red Activator, Sun Medical, Moriyama, Japan) to the surface for 30 s using a sponge applicator (Sun Medical, Moriyama, Japan), after which the acid was fully removed by rinsing with an air-water spray. Super-Bond/Clear fluoride-free self-cure adhesive resin (4-META/MMA-TBB, Sun Medical) was prepared by mixing the liquid, catalyst, and powder according to the manufacturer’s instructions. The mixed adhesive was delivered to the bracket base with a fine brush. The bracket was then promptly placed at the center of the etched enamel surface and held firmly in position until setting was complete, following the time specified by the manufacturer. The adhesive was then allowed to chemically cure for 8 min.

### 2.6. Mold Preparation

Crown separation was achieved by cutting each tooth at the cementoenamel junction, allowing the coronal portion to be accommodated within the cylindrical acrylic mold. The crown was then embedded in a mold made of autopolymerizing acrylic resin (Unifast II, GC Dental Industrial Corp., Tokyo, Japan) with the labial enamel face left exposed and parallel to the direction of the applied shear force. This embedding procedure was performed to firmly fix each specimen in a standardized position during SBS testing, guaranteeing that the loading direction remained consistent with the plane of the bracket base throughout testing. The crown was embedded in autopolymerizing acrylic resin using a circular plastic mold.

### 2.7. SBS

**Figure 2:**
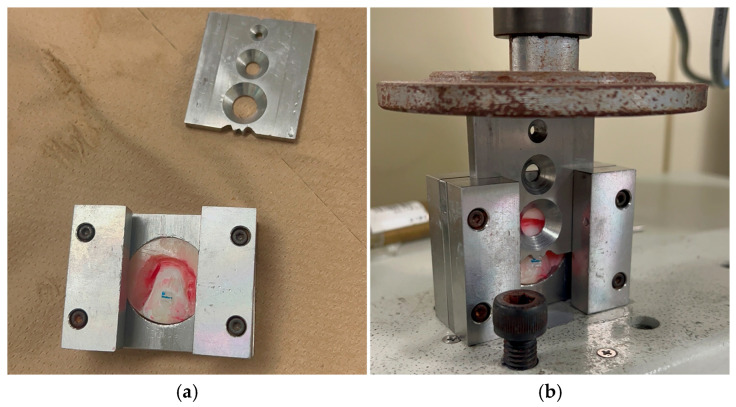
Experimental setup for SBS testing. (**a**) Bovine incisor crown embedded in autopolymerizing acrylic resin, placed within the SBS testing jig. (**b**) The testing jig with the embedded specimen mounted in the universal testing machine (Autograph AGS-J; Shimadzu, Tokyo, Japan), with the cutting blade positioned between the bracket wing and the bracket base, perpendicular to the bracket slot, applying a shear force parallel to the bracket base at a crosshead speed of 1 mm/min.

The acrylic-embedded specimens were subsequently loaded into a universal testing machine (Autograph AGS-J; Shimadzu, Tokyo, Japan). such that shear loading was delivered in a direction parallel to the bracket base at a crosshead displacement rate of 1 mm/min. Debonding loads were captured in Newtons and subsequently converted to MPa by dividing each recorded force by the bonding area of the bracket base (12.00 mm^2^). Before mechanical testing, all bonded specimens were submerged in deionized water at 37 °C for 24 h to ensure complete curing of the adhesive resin. The experimental setup for SBS testing is illustrated in Figure 2.

### 2.8. Adhesive Remnant Index (ARI)

Following bracket removal, both the enamel surfaces and the debonded bracket bases were inspected with a stereomicroscope (SMZ1000; Nikon, Tokyo, Japan) connected to a CCD camera (DS-Fi1; Nikon, Tokyo, Japan) to classify the type of failure. Failure type was further characterized by applying the ARI [42], a four-grade classification system defined as follows:

0 = No adhesive detected on the tooth surface.

1 = Adhesive remaining less than half of the bonded area.

2 = Adhesive remaining more than half of the bonded area.

3 = The bonded area entirely remained by residual adhesive.

### 2.9. Statistical Analysis

All statistical analyses were performed using IBM SPSS Statistics software, Version 27.0 (IBM Corp., Armonk, NY, USA), with the significance level set at α = 0.05. The normality of the SBS data distribution was assessed using the Shapiro–Wilk test, which confirmed a non-normal distribution (*p* < 0.05); consequently, non-parametric tests were applied. For SBS, Dunn’s test with Bonferroni correction was used for pairwise comparisons to control for Type I error across multiple comparisons. LD was analyzed using the Mann–Whitney U test (*p* = 0.631). ARI scores, being ordinal data, were assessed applying the Kruskal–Wallis test (*p* = 0.0127), followed by Dunn’s test with Bonferroni correction for post hoc pairwise comparisons.

## 3. Results

**Figure 3:**
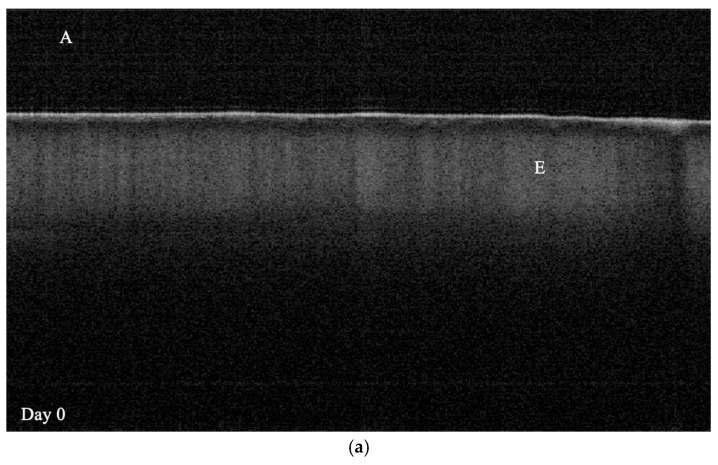

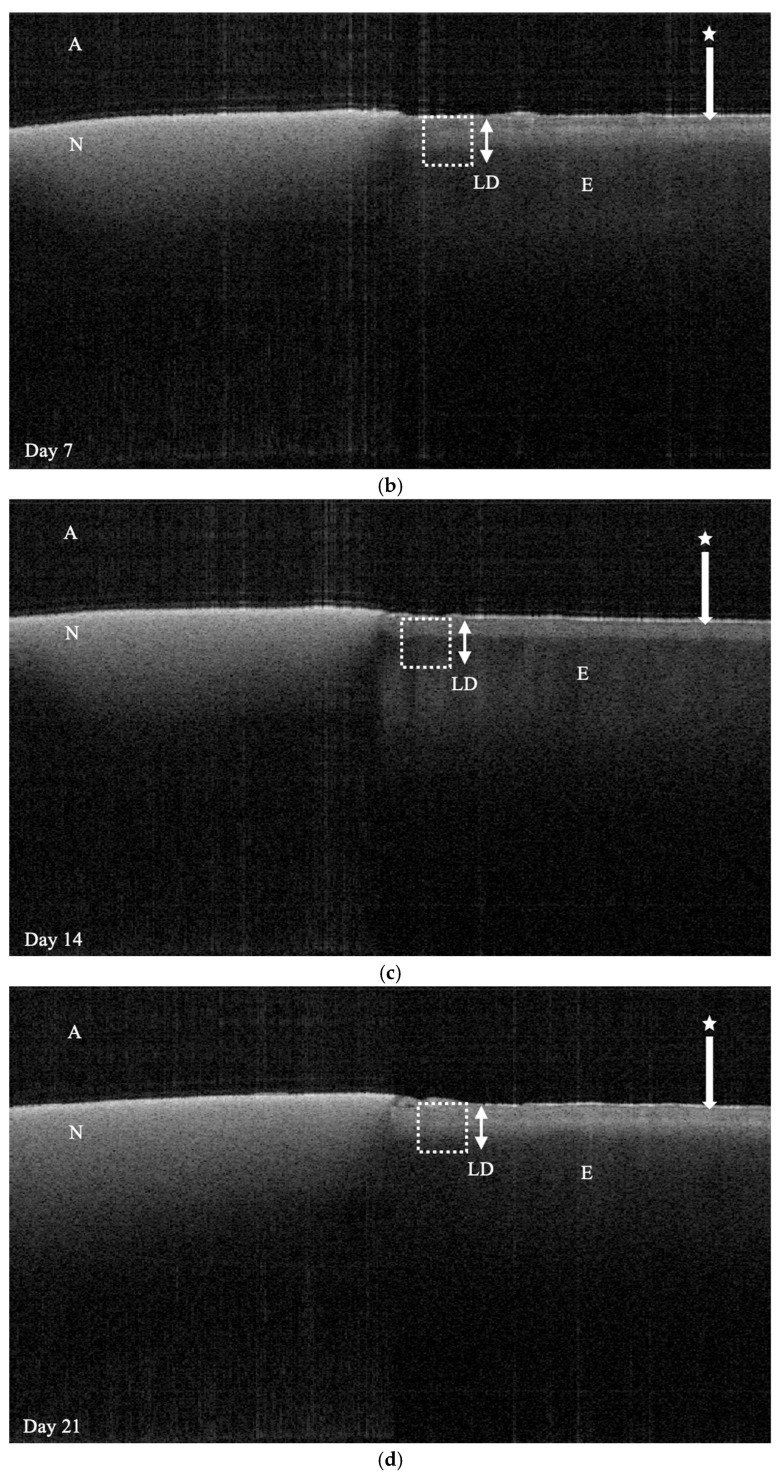
SS-OCT images showing gradual increases in demineralization depth from day 7. The visible boundary was used to define the demineralization area (indicated by a star). A region of interest (ROI, shown as a dotted box) was established immediately adjacent to the nail varnish border for lesion depth measurement. A, E, N, and LD denote air medium, enamel, nail varnish, and lesion depth, respectively. (**a**) Day 0, (**b**) Day 7, (**c**) Day 14, and (**d**) Day 21.

Signal intensity variations associated with demineralization are illustrated in Figure 3.

**Figure 4:**
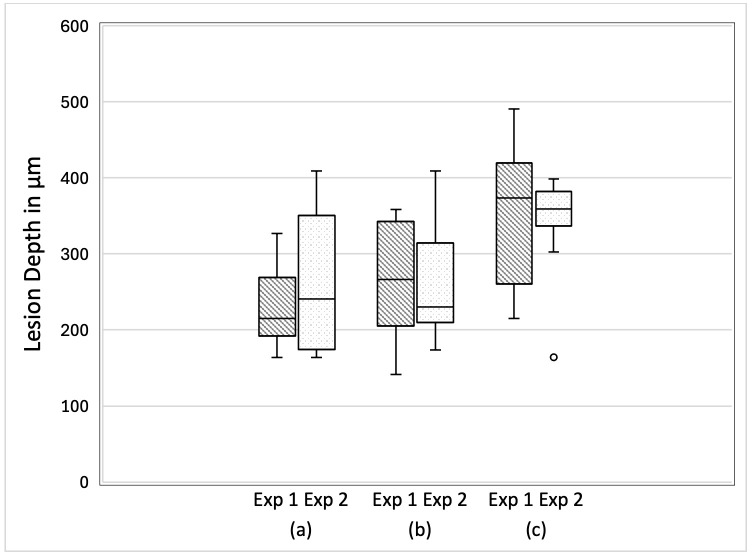
SS-OCT analysis of LD (µm) during the demineralization period. In the box-and-whisker plots, the median is represented by a midline bisecting the box, while the interquartile range (25th–75th percentiles) reflects the distribution of LD values measured within the region of interest (ROI). The whiskers indicate the minimum and maximum values. Dots (○) represent outlying data points, identified as values exceeding 1.5 times the interquartile range (IQR) beyond either the upper or lower quartile boundary. LD values were obtained directly from the scaled SS-OCT images without refractive index correction. (a) Day 7, (b) Day 14, and (c) Day 21.

Lesion depth showed no statistically significant difference between the Exp1 and Exp2 groups. The progression of LD across the demineralization period is depicted in Figure 4.

**Figure 5:**
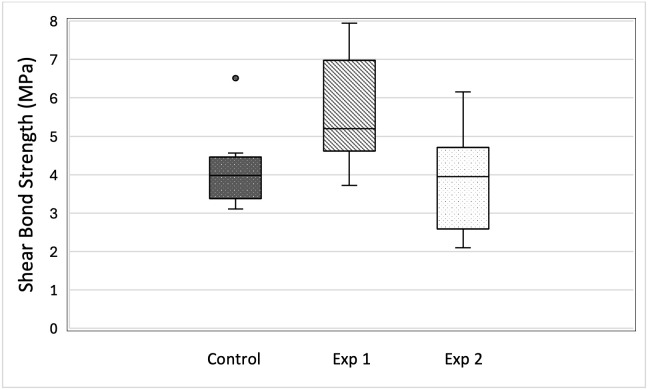
Distribution of SBS measurements among the three groups displayed as box-and-whisker plots (MPa): control, demineralized enamel pretreated with ICON^®^ resin infiltrant (Exp1 group), and demineralized enamel without pretreatment (Exp2 group). The rectangular box spans the interquartile range (25th to 75th percentiles), the central horizontal line marks the median, and the extending whiskers indicate the full data range from minimum to maximum. Dots (○) represent outliers, defined as values more than 1.5 times IQR, beyond the upper or lower quartile.

The SBS results are presented in Figure 5 and Table 1.

Among the three groups, Exp1 yielded the highest median SBS while Exp2 recorded the lowest. The single highest SBS measurement was also obtained within Exp1, and the absolute minimum fell within Exp2. Statistically significant SBS differences were found between the control and Exp1 groups and between the Exp1 and Exp2 groups. No statistically significant difference in SBS was detected between the control and Exp2 groups.

**Figure 6:**
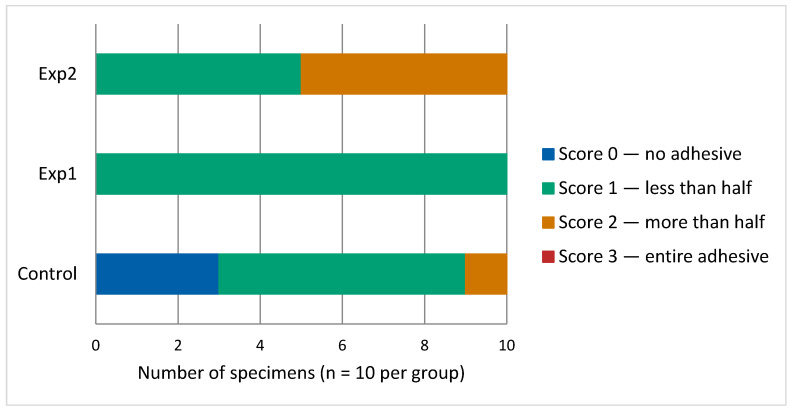
Distribution of ARI scores among the three experimental groups. Each bar represents 100% of the specimens (*n* = 10 per group). Score 0: tooth surface entirely free of adhesive; Score 1: adhesive remaining less than half the bonded area; Score 2: adhesive remaining more than half the bonded area; Score 3: complete adhesive layer retained on the tooth surface.

Regarding the ARI scores, the Exp2 group displayed the highest ARI score, whereas the control group demonstrated the lowest ARI score. No significant difference was identified between the control and Exp1 groups; in contrast, statistically significant differences were confirmed between the control and Exp2 groups. The distribution of ARI scores across the three groups is presented in Figure 6.

The Exp1 group showed a uniform failure pattern, with all specimens (10/10) recording Score 1, signifying that less than half of the adhesive layer was present on the enamel following bracket detachment. The control group demonstrated predominantly Score 1 (6/10), with Score 0 recorded in 3 specimens and Score 2 in one specimen. The Exp2 group showed a mixed failure pattern, with equal distribution between Score 1 (5/10) and Score 2 (5/10) and greater amounts of residual adhesive on the enamel surface relative to both the control and Exp1 groups.

## 4. Discussion

The present laboratory study was conducted to examine how pre-treatment with ICON^®^ resin infiltrant affects the SBS of orthodontic brackets placed on demineralized enamel. No significant difference in LD was observed between groups. Resin infiltration significantly improved SBS, and ARI scores differed significantly between the control and Exp2 groups.

As shown in Figure 3a–d, the demineralization depth increases over time. Under SS-OCT, structurally intact enamel exhibits near transparency when imaged in the near-infrared spectrum, whereas demineralized enamel exhibits increased signal attenuation [43,44]. SS-OCT detects enamel demineralization as a bright area with increased backscatter owing to mineral loss [45]. In grayscale SS-OCT images, a clear visual interface between the dark and bright regions has been reported to correspond to LD [46]. Signal attenuation increases with lesion depth because the effective penetration of SS-OCT is limited to a range of only a few millimeters below the tooth surface. The area of demineralization within the ROI was then computed from the identical SS-OCT images used for both Exp1 and Exp2 analysis. Because images obtained before the demineralization protocol began represented a baseline of zero mineral loss, lesion depth was not calculated from day 0 data in any of the three groups. The optical LD values (µm) shown in Figure 4 were calculated by dividing the measured demineralized cross-sectional area by the fixed ROI width of 333.3 µm. The interpretation of the true LD requires consideration of the enamel refractive index (1.631) [47].

Demineralized enamel has been consistently associated with reduced SBS in orthodontic brackets [25]. In contrast, one study reported no statistically significant SBS difference between intact and demineralized enamel substrates [48]. Phosphate mapping of the enamel specimens suggested that a thin surface layer remained intact overlying a subsurface region with reduced phosphate content, coinciding with the main body of the carious lesion [49]. Although the inner enamel layers had undergone mineral loss, it is possible that the preserved surface layer allowed penetration of some adhesives after acid etching. This finding may partly explain why SBS did not differ markedly from sound enamel. This observation is consistent with that of previous literature, indicating that bond strength is highly dependent on enamel surface topography [50].

Among the several interventions, resin infiltration was demonstrated to provide significantly higher SBS than other treatments, such as casein phosphopeptide–amorphous calcium phosphate or fluoride varnish [51]. Resin infiltration penetrates the porous structure of mineral-depleted enamel, occluding the intercrystalline channels through the formation of a cross-linked polymer network distributed throughout the lesion body. This polymer network micromechanically stabilizes the enamel structure and blocks the residual enamel prisms, thereby halting further demineralization [52,53,54]. Owing to their low viscosity, the microscopic spaces between the enamel and the spaces between enamel crystals become occupied by the infiltrating resin, thereby establishing a continuous diffusion barrier within the demineralized zone. This process reinforces the demineralized enamel, increasing its hardness and mechanical strength [53]. Consistent with these findings, multiple investigations have documented that resin infiltration treatment is capable of restoring bracket bond strength to values comparable with, or in some cases exceeding, those measured on sound enamel [25,55,56,57,58].

In view of the ICON formulation as a triethylene glycol dimethacrylate-based methacrylate resin infiltrant [59], polymerization in an oxygen-rich environment may result in a thin oxygen-inhibited surface layer, which is a well-recognized feature of methacrylate resin systems [60]. To date, no published studies have demonstrated a specific chemical bonding mechanism at the bonding zone between the set ICON infiltrant and the 4-META/MMA-TBB resin component of Super-Bond. Accordingly, the observed bonding effectiveness is more appropriately attributed to the micromechanical retention afforded by the modified enamel substrate rather than to the confirmed chemical copolymerization. In this study, resin infiltration of demineralized enamel significantly improved the SBS compared with that of untreated demineralized enamel. The median SBS for Exp1 was 5.2 MPa, with Exp2 producing a lower value of 3.9 MPa. A median SBS of 4.0 MPa was obtained in the control group.

The ARI scores further supported these findings. Statistically significant between-group ARI differences were confirmed for the control versus Exp2 comparison and for the Exp1 versus Exp2 comparison.An increase in ARI score reflected a larger proportion of adhesive retained on the enamel upon bracket detachment. The Exp2 group exhibited the highest ARI scores, suggesting fracture occurring within the body of the adhesive resin itself rather than at the junction between the adhesive and the enamel surface; however, this was not associated with increased SBS. This observation suggests that the ARI represents the location of bond failure [61,62]. No significant correlation was observed between debonding strength and ARI scores [63]. Traditional orthodontic bond-strength studies may not accurately reflect the true likelihood of failure within a bonding system. Moreover, conclusions regarding the strength of individual components based solely on observed fracture locations should be interpreted with caution because failure may initiate from stress concentrations rather than from the uniform stress distribution assumed in standard tests [64].

Conversely, lower ARI scores in the control and Exp1 groups suggest that failure predominantly occurred at the boundary between the adhesive and the enamel. This failure pattern is clinically favorable because it leaves minimal adhesive material on the tooth, reducing the time and effort required for surface clean-up after debonding [65,66].

Although all the experimental procedures were carefully standardized, this study has several limitations. First, the use of bovine incisors as a surrogate for human dentition may limit the direct applicability of these findings to clinical settings. Second, only one adhesive system (Super-Bond/Clear, 4-META/MMA-TBB, Sun Medical) was evaluated; future studies should investigate other clinically used adhesive systems. Third, only plastic brackets were tested, whereas metal and ceramic brackets are more commonly used clinically [67,68]. Fourth, Super-Bond/Clear is a hand-mixed self-cure adhesive that differs from light-cured adhesives commonly used in clinical orthodontic practice. Fifth, demineralization was induced prior to bracket bonding, which may not fully replicate the clinical scenario where demineralization typically occurs around bonded brackets rather than beneath them. Finally, this study was conducted in vitro, and in vivo investigations will be required to confirm and extend these findings under the complex conditions of the oral environment.

## 5. Conclusions

Pretreatment of demineralized enamel with ICON^®^ resin infiltrant significantly improved the SBS recorded for brackets bonded to demineralized enamel compared with those that received no prior treatment. Additionally, resin infiltration resulted in a more favorable ARI score distribution, suggesting a clinically advantageous failure pattern at the enamel-adhesive interface. The evidence obtained from this study supports that ICON^®^ resin infiltration may represent a promising approach for bracket bonding on demineralized enamel in vitro.

## Figures and Tables

**Table 1:** Median, IQR, maximum and minimum shear bond strength (SBS) measurements recorded across the three study groups (MPa) (*n* = 10).

Group	Median	IQR	Min.	Max.
control	4.0	1.0	3.1	6.5
Exp1	5.2	1.7	3.7	7.9
Exp2	3.9	1.8	2.1	6.2

## Data Availability

The original contributions presented in this study are included in the article. Further inquiries can be directed to the corresponding author.

## Abbreviations

The following abbreviations are used in this manuscript:

ARI	Adhesive remnant index
WSL	White spot lesion
SBS	Shear bond strength
LD	Lesion depth
SS-OCT	Swept-source optical coherence tomography
ROI	Region of interest
IQR	Interquartile range
